# Anatomical and morphological variations associated with *in vitro* micrografting in olives, using clonal rootstocks

**DOI:** 10.3389/fpls.2026.1876003

**Published:** 2026-07-10

**Authors:** Nourhene Jouini, Jorge Canhoto, Elsa Baltazar, Tércia Lopes, Irene Granata, Maria Antonietta Germanà, Francesco Paolo Marra, Tiziano Caruso, Annalisa Marchese

**Affiliations:** 1 Department of Agricultural, Food and Forestry Sciences, University of Palermo, Palermo, Italy; 2 Center for Functional Ecology, Associate Laboratory Terra, Department of Life Sciences, University of Coimbra, Coimbra, Portugal

**Keywords:** grafting, micropropagation, microscopy, *Olea europaea*, vascular differentiation

## Abstract

Micrografting is a powerful biotechnological tool for the micropropagation of fruit trees and can be applied to clonal propagation of recalcitrant species, virus eradication, pathogen management, early screening of scion/rootstock compatibility, and breeding. In olive, however, this technique remains poorly explored, particularly regarding grafting onto fully clonal rootstocks. In this study, we developed an *in vitro* micrografting (IVM) protocol based entirely on clonally propagated material. Two cultivars: ‘Galega’ and ‘Arbequina’, and two genotypes: genotype 26 (G26), and Genotype 35 (G35), were selected based on their vigor and rooting ability. IVM was optimized by testing different combinations of scion and rootstock length and diameter. Morphological variations, including callus formation, wound healing, and vascular connection, were evaluated in both homografting (Galega) and heterografting combinations. Histological analyses were performed at 7, 14, 21, 30, 45, and 60 days after micrografting (DAM) to characterize graft union development. Wounding occurred as early as 7 DAM, while between 14 and 21 DAM, parenchymatous cells proliferated and differentiated into tracheid cells, indicating the wound healing process. By 30–45 DAM, vascular continuity was re-established, confirming successful graft formation. Overall, these results provide a detailed temporal and anatomical characterization of graft union development and support the optimization and scaling-up of IVM for nursery production. Furthermore, this approach may contribute to sustainable strategies against emerging threats such as *Xylella fastidiosa* and may allow modulation of vegetative vigor through rootstock selection.

## 1 Introduction

The Olive tree (*Olea europaea* L.) is one of the oldest cultivated tree species and represents a crop of major socio-economic, cultural, nutritional, and environmental relevance worldwide (Fabbri, 2023). The *Olea* L. genus includes more than 30 species (Green, 2002), but *Olea europaea* L. is the only species widely cultivated for fruit and oil production, with distribution across Europe, Asia, Oceania, and Africa. The Mediterranean basin, characterized by warm, dry summers and mild, wet winters, accounts for approximately 98% of global olive production, highlighting the importance of this crop in the regional economy and driving the need for innovative propagation strategies (Fraga et al., 2021).

Despite extensive attempts to enhance self-rooting success for olive’s vegetative propagation, many cultivars remain highly recalcitrant, making grafting on seedlings the only solution for asexual reproduction in the nursery industry (Fabbri et al., 2009; Lambardi et al., 2012). Grafting is a well-established horticultural practice that involves the physical union of two plant parts, namely the scion and the rootstock, which subsequently develop a functional vascular connection (Mudge et al., 2009; Feng et al., 2024). The formation of the graft union is a complex biological process involving cell division, proliferation, and differentiation, ultimately leading to the restoration of vascular continuity (Pina and Errea, 2005).

In olive, grafting onto seedling rootstocks in the nursery, or tree suckers in the field, is commonly used; however, this approach is labor-intensive, season-dependent, and requires long production times before field establishment (Lambardi et al., 2012, Lambardi et al., 2023). Therefore, the development of more efficient and standardized grafting systems, particularly involving selected clonal rootstocks, is essential to accelerate the availability of elite cultivars with improved agronomic performance and abiotic and biotic stress tolerance. In this context, micropropagation represents a powerful tool for the large-scale production of olive plant material, enabling the generation of uniform, certified pathogen-free plants.

Over the past decades, plant tissue culture has been applied in olive, and it has achieved many innovations and protocol improvements, including the use of new compounds, new techniques and methods, and a combination of *in vitro* and ex vitro production in nurseries to provide the best plantlet quality (Micheli et al., 2025). Following the development of *in vitro* plant tissue culture in the middle of the 20th century, the first attempts to combine grafting with tissue culture, known as *in vitro* micrografting, were reported in *Ivy* and *chrysanthemum* during the 1950s (Doorenbos, 1954; Holmes, 1956). This approach was later refined by Navarro et al. (1975), who demonstrated its effectiveness in virus elimination in *Citrus* species. In olive, IVM was first described by Revilla et al. (1996), who grafted *in vitro* shoots onto seedling-derived rootstocks, highlighting its potential for rejuvenation of mature tissues. Subsequently, Troncoso et al. (1999) applied *in vitro* cleft grafting to investigate the anatomical processes underlying graft union formation using embryos germinated *in vitro*. More recently, IVM protocols have been optimized for specific olive cultivars, such as ‘Zard’ (Farahani et al., 2011) and ‘Kalamata’ (Sayed et al., 2024), demonstrating high efficiency and genetic stability of regenerated plants. These studies underline the potential of IVM as a rapid and reliable system for evaluating scion–rootstock compatibility and physiological interactions. In a recent study, Montilon et al., 2026 used IVM in olive together with the thermotherapy to eliminate olive leaf yellowing-associated virus (OLYaV). This recent work highlighted the efficiency of this method to produce virus free plants. However, in all previous studies, *in vitro* grafting has been performed using seedling-derived rootstocks, while scions were obtained from *in vitro*-proliferated shoots. To date, no studies have reported the use of fully clonal rootstocks derived from *in vitro*–propagated cuttings for IVM in olive (Farahani et al., 2011; Sayed et al., 2024; Montilon et al., 2026). In the present study, we established an *in vitro* micrografting protocol based entirely on clonal material, using rootstocks obtained from multiplied and rooted cuttings *in vitro*. Different experimental conditions were tested to identify the optimal combinations of scion and rootstock size (length and diameter) for successful graft establishment. This system provides a valuable platform for the detailed analysis of histological events occurring during graft union formation over time. Furthermore, the proposed approach may represent a key step toward the development of scalable propagation systems, particularly in the context of emerging threats such as *Xylella fastidiosa*, by enabling the rapid multiplication of selected, potentially certified, resilient plant material against biotic and abiotic stresses.

## 2 Materials and methods

### 2.1 Plant materials

The experiments were conducted using four olive genotypes propagated *in vitro*: ‘Galega’, ‘Arbequina’, G26’, and ‘G35’. The cultivars ‘Arbequina’ and ‘Galega’ were selected for their contrasting rooting behaviors, with ‘Arbequina’ exhibiting high rooting ability and ‘Galega’ being more recalcitrant. The genotypes ‘G26’ and ‘G35’, derived from the self-pollination of the cultivar ‘Koroneiki’ developed by the Department of Agricultural, Food and Forest Sciences ‘SAAF’ at the University of Palermo (Marchese et al., 2016; Granata et al., 2025), were included due to differences in vigor, with ‘G26’ showing high vigor, while ‘G35’ showing low vigor.

### 2.2 IVM protocol

*In vitro* micrografting assays were carried out using micro-propagated olive cuttings from mother trees of the above-mentioned cultivars, applying ‘V’ grafting method. After establishment and multiplication as described by Jouini et al. (2024) and Granata et al. (2023), micro-scions and micro-rootstocks were multiplicated in Rugini Olive medium (OM) supplemented with 10 µM Zeatin, 7 g L^-1^ agar, 30 g L^-1^ mannitol, and pH was adjusted at 5.8 prior to autoclaving. Then, subculturing was performed every 3–4 weeks to ensure sustained growth and development of the cultures under their respective experimental conditions. Micro-rootstocks were rooted *in vitro*, in OM supplemented with 2 mg L^-1^ of IBA and 2 mg L^-1^ of NAA. These cultures were incubated for a period of 30 days in a controlled growth chamber set at 25 °C under a photoperiod (16/8) light-dark.

For scion (SC) preparation, the apical portion of each plantlet, including the shoot meristem, was fully defoliated, preserving the apical and the axillary meristems. Rootstocks (RT) were prepared by removing the basal portion and eliminating all leaves and axillary meristematic regions. Initial trials focused on homo-grafting of ‘Galega’ to optimize the IVM protocol. Two preliminary experiments were performed: the first assessed stem diameter compatibility among cultivars the second tested the effect of scion length and rootstock length (1, 2, and 3 cm) on graft success. In both experiments, length and diameters were measured conventionally and then pictures of both parts were taken using digital camera and then measured again using ImageJ software. Following protocol optimization, graft compatibility was evaluated through reciprocal micrografting among four olive cultivars (**Table 1**), selected for their diverse physiological traits and based on the available plant material. Graft success was assessed by callus formation at the graft union, wilting symptoms, and emergence of new shoots from the scion, which can be distinguished approximately 15 days post IVM. Each treatment consisted of 45 explants distributed across three biological replicates of 15 explants each.

**Table 1:** Combinations (scion × rootstock) of micrografting between cultivars.

Rootstock	Scion			
	Galega (G)	Arbequina (AR)	G26 (26)	G35 (35)
Galega	×	×	×	
Arbequina	×	×		
G26	×		×	×
G35			×	×

### 2.3 Histological analysis

Histological analyses were performed at various intervals following the initiation of IVM, ranging from one to eight weeks precisely at 7, 14, 21, 30, 35, 45, and 60 days after micrografting (DAM). These points were selected to comprehensively examine the developmental progression of the vascular connection between the rootstock and scion following the grafting process. The study initially focused on the homo-grafting model (Galega as scion and as rootstock), to establish a reproducible reference model for *in vitro* graft union development in olive, enabling protocol optimization and the evaluation of healing dynamics independently of intergenotypic compatibility effects. After that, comparisons with the heterografting combinations summarized in **Table 1** were conducted. Four samples were analyzed at each point. Graft union samples, taken 1 cm above and below the junction, were initially fixed in formalin–acetic acid–alcohol (FAA) solution for seven days. Samples were then dehydrated through a graded ethanol series (50%, 70%, 90%, 95%, and absolute ethanol), cleared in Clear-Rite 3 ™ solution by Epredia, and embedded in paraffin. Thin sections (8–10 µm thick) were obtained using a rotary microtome and stained according to the specific histological analysis required. Sections stained with Fast Green alone were immersed in the staining solution for 2 min, whereas sections stained with Safranin alone were immersed for 5 min. For double-staining procedures, sections were first stained with either Safranin or Toluidine Blue for 3 min, rinsed with distilled water, and subsequently counterstained with Fast Green for 2 min. In addition, a mixed staining solution consisting of Safranin and Toluidine Blue (1:1;v/v) was prepared, and sections were stained for 5 min. Following staining, the sections were rinsed with distilled water. The figures presented in this work were selected from multiple observations based on the quality of staining, tissue preservation, and image resolution to ensure optimal visualization of the anatomical features described. Observations were conducted using a binocular microscope (Nikon ECLIPSE Ci), and images were captured with NIS-Elements software, version 4.6B.

### 2.4 Data analysis

Grafting success rate was calculated as the proportion of successfully grafted plants relative to the total number of plants grafted. Data were analyzed statistically using R Studio software (Posit team, 2023). Percentage data were arcsine-transformed before analysis. Significance was determined by analysis of variance (ANOVA), and mean differences were compared using the LSD test.

## 3 Results

### 3.1 *In vitro* micrografting

#### 3.1.1 Effect of the scion and rootstock length on the graft success

**Figure 1:**
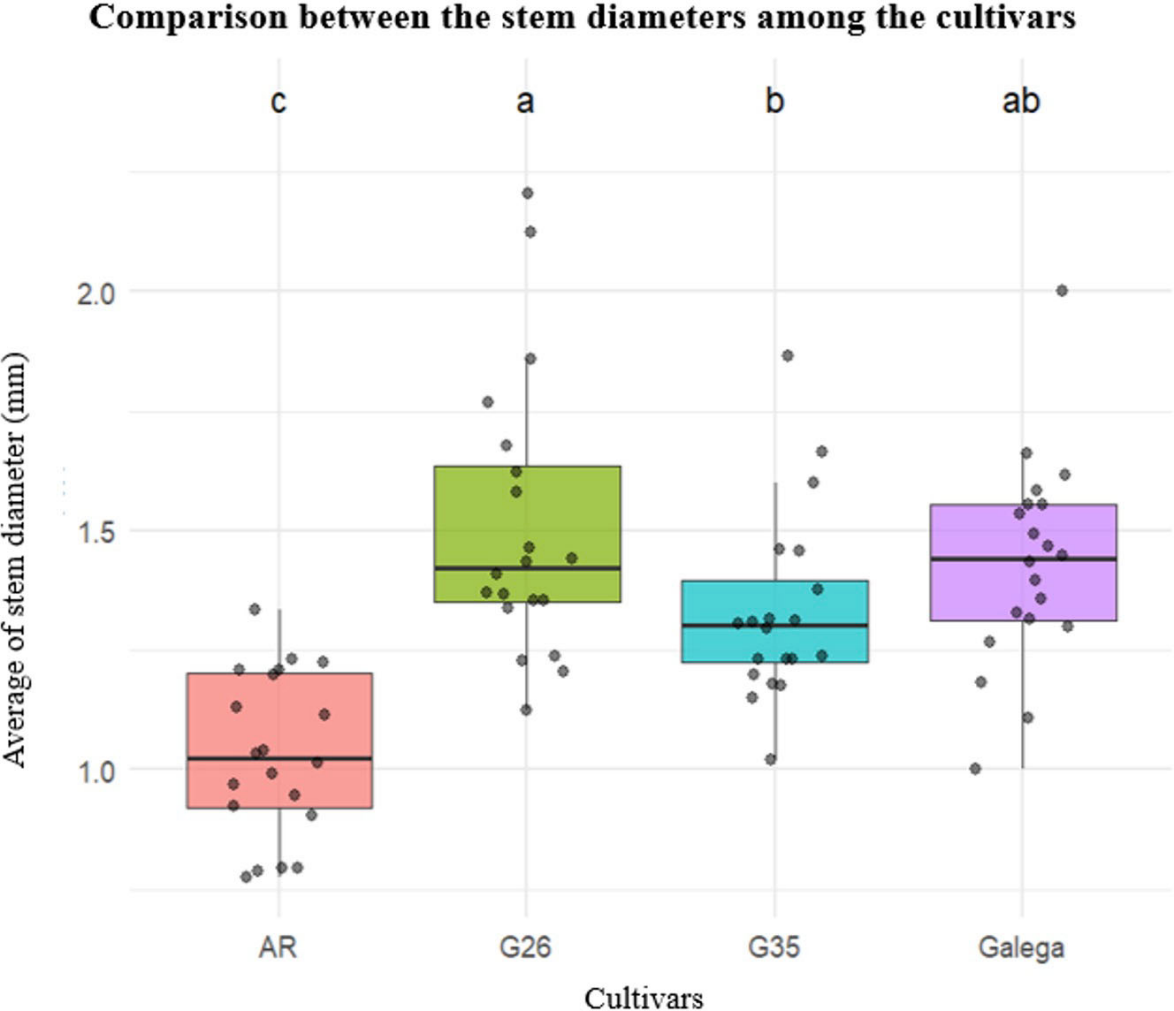
Comparison between the stem diameters of the different cultivars. (Parameters submitted to one-way ANOVA analyses separately; Letters a, b and c, showed the difference of means among media results present after LSD test (α < 0.001)).

To optimize the IVM protocol, experiments were initially conducted to evaluate the effect of different length ratios between micro-scions and micro-rootstocks. Homo-grafting of the ‘Galega’ cultivar was used to assess the length of 1, 2, and 3 cm. The results, summarized in **Table 2**, indicate that the selection of appropriate lengths strongly influences grafting. Optimal success rates (100%), with no graft failure, were achieved when the scion was 1 cm long and rootstock was 1–3 times longer. In contrast, when the scion was 2–3 times longer than the rootstock, grafting success declined to 75% - 90%, with a corresponding increase in graft collapse. These results emphasize the critical importance of adopting the appropriate length ratio between the scion-to-rootstock for a successful *in vitro* micrografting. Subsequent measurements of stem diameter across cultivars revealed significant differences (**Figure 1**), as determined by ANOVA with LSD grouping. The analysis identified three distinct groups: ‘a’ for G26, ‘b’ for G35, and ‘c’ for Arbequina. Galega was classified as ‘ab’, indicating that its stem diameter differed significantly from Arbequina but not from the other cultivars.

**Table 2:** Effect of the scion and rootstock on the graft success rate.

Length of rootstock (cm)	Length of scion (cm)		
	1	2	3
1	100 % ^(a)^	25 % ^(d)^	10 % ^(e)^
2	100 % ^(a)^	90 % ^(b)^	40 % ^(c)^
3	100 % ^(a)^	100 % ^(a)^	25 % ^(d)^

Values submitted to one way ANOVA analyses separately; Letters a, b, c, d and e showed the difference of means among media results present after LSD test (α< 0.001).

#### 3.1.2 IVM to test the compatibility between cultivars

**Figure 2:**
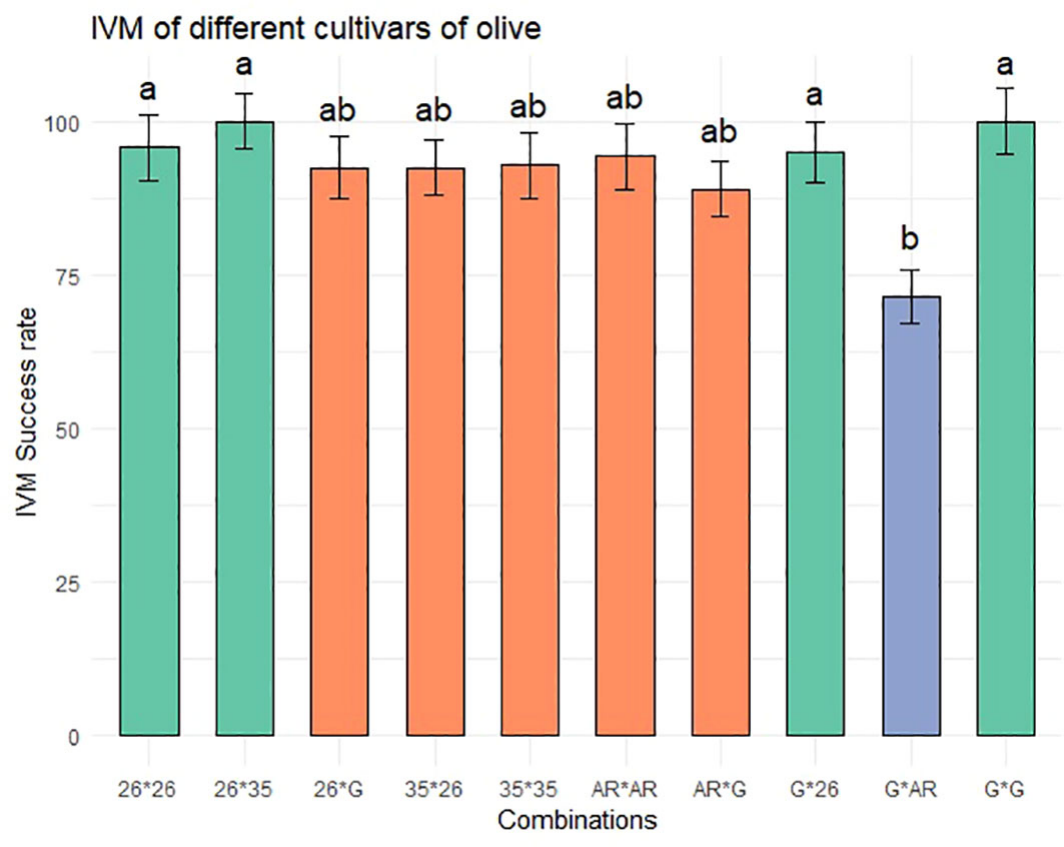
Success rate of the different IVM combinations among cultivars.

Following the optimization of the IVM protocol, experiments were conducted to evaluate heterografting compatibility among different cultivar combinations. Grafting success rates showed limited variation depending on the scion–rootstock pairing. **Figure 2**, illustrates the compatibility of reciprocal graft combinations among ‘Galega’, ‘Arbequina’, ‘G26’, and ‘G35’.When ‘Galega’ was used as rootstock, the highest success rate was observed in homografting (100%), while grafting with ‘Arbequina’ and ‘G26’ scions resulted in slightly lower success rates (89% and 92.5%, respectively), with no statistically significant differences among treatments.

‘Arbequina’ used as rootstock exhibited a more selective behavior, showing significantly reduced compatibility when grafted with ‘Galega’ (71.45%). The ‘G26’ rootstock performed consistently well in both homo- and heterografting combinations, with success rates exceeding 95% when grafted with itself or ‘Galega’, and slightly lower values (92.5%) when combined with ‘G35’; all treatments belonged to the same statistical group, indicating overall compatibility. Similarly, G35’ used as rootstock showed high compatibility in homografting (100%) and when grafted with ‘G26’ (92.85%).

Overall, these results demonstrate a high degree of graft compatibility among the tested genotypes, except for the ‘Galega’ × ‘Arbequina’ combination, which exhibited significantly lower success rates classified in group b, as shown in **Figure 2**.

### 3.2 Morphological analysis following IVM

**Figure 3:**
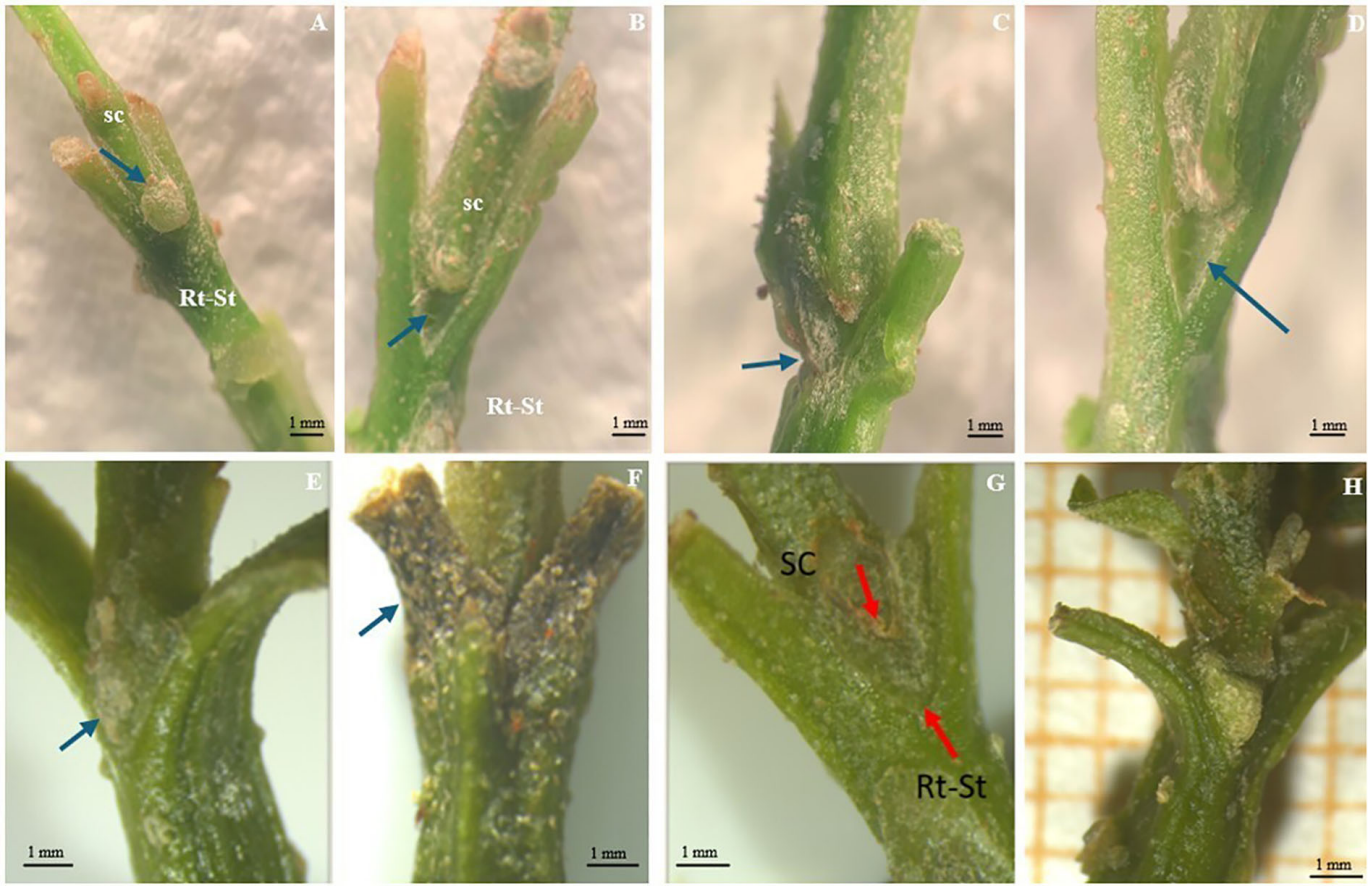
Morphological alterations observed at the graft junction during the healing process. **(A)** Globular callus formation at 30 days after micrografting. **(B, C, E)** Different patterns of callus formation at 30 DAM. **(D)** Early callus formation at 10 DAM. **(F)** Oxidation at the graft union, indicating graft failure. **(G)** Callus bridge becoming more compact and rigid at 60 DAM.

To assess early morphological changes at the graft interface, the IVM process was initially evaluated through stereomicroscopic observations focused on callus formation at the graft junction. Callus development was used as a primary indicator of graft union formation and tissue compatibility. Within 10 days of post-micrografting, the first signs of callus initiation were evident (**Figure 3D**). 15 DAM, proliferating callus gradually expanded to form a distinct callus bridge, physically connecting the scion and rootstock within the first three weeks after grafting (**Figure 3**). The expansion and morphology of callus at the graft junction exhibited variation. For example, (**Figure 3A**) shows a globular callus formed 30 days after micrografting (DAM), while other proliferation patterns are illustrated in (**Figures 3B, C, E**). This callus bridge increased in both width and density over time, reflecting progressive tissue differentiation and early stages of vascular reconnection, as observed in (**Figure 3G**) at 60 DAM. Callus formation was consistently observed in both homo and heterografting, with no apparent differences, indicating that this response represents a general morphological feature for IVM rather than a genotype-specific trait (**Figure 3**).

### 3.3 Histological analysis

**Figure 4:**
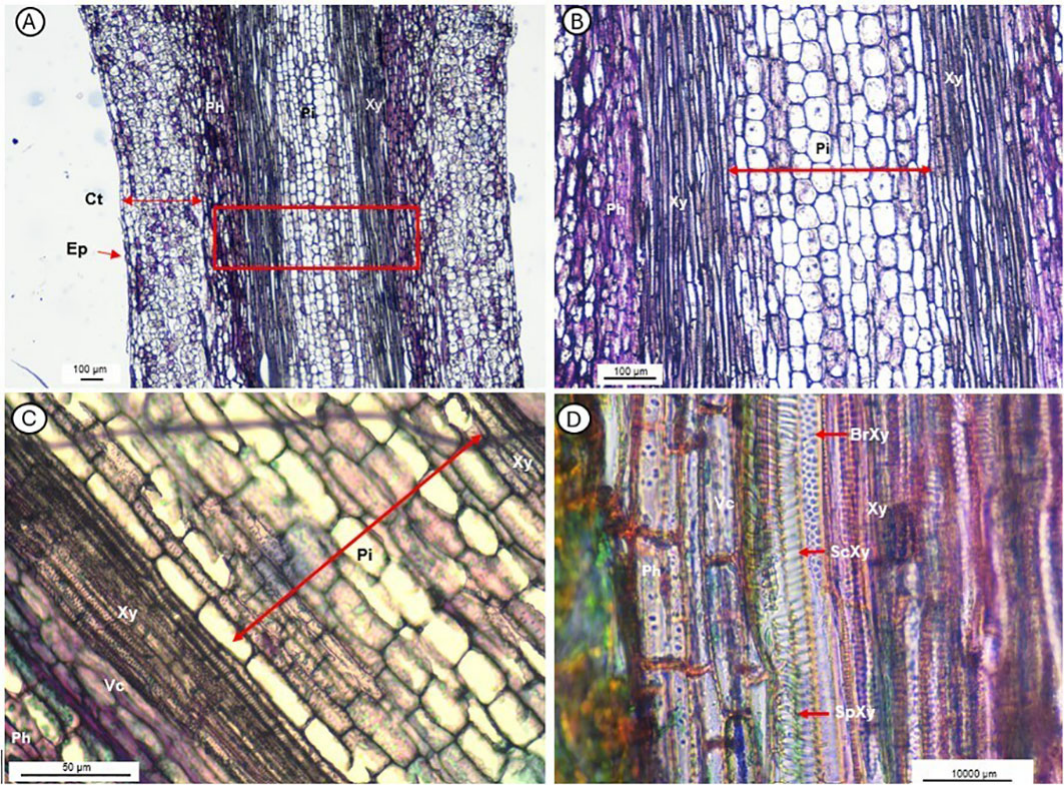
Longitudinal section of a stem of *O. europaea* to show the arrangement of tissues before micrografting. Stems were from plants grown *in vitro* for two years and after several subcultures. **(A)** General observation of the section showing the different tissues. **(B)** Detail of the vascular tissues and pith (red area in A). **(C)** Higher magnification of the vascular tissues and pith. **(D)** Detail of xylem cells easily identified by secondary wall thickenings and pits. BrXy: bordered xylem cells, Ct, Cotrex; Ep, Epiderm; Ph, Phloem; Pi, Pith; ScXy, Scalariform xylem vessels; SpXy, Spiral xylem vessels; Vc, Vascular cambium; Xy, Xylem.

Following the identification of morphological changes, histological analysis was conducted weekly post-IVM to better understand the healing process in both homo- and heterografts. Initially, longitudinal histological sections were prepared from intact *in vitro* olive stems of the cultivar ‘Galega’ to serve as a reference for post-IVM events. As shown in (**Figures 4A, B**), the tissue of an intact olive stem is well organized, with distinct xylem vessel types clearly observable, including scalariform spiral and bordered.

**Figure 5:**
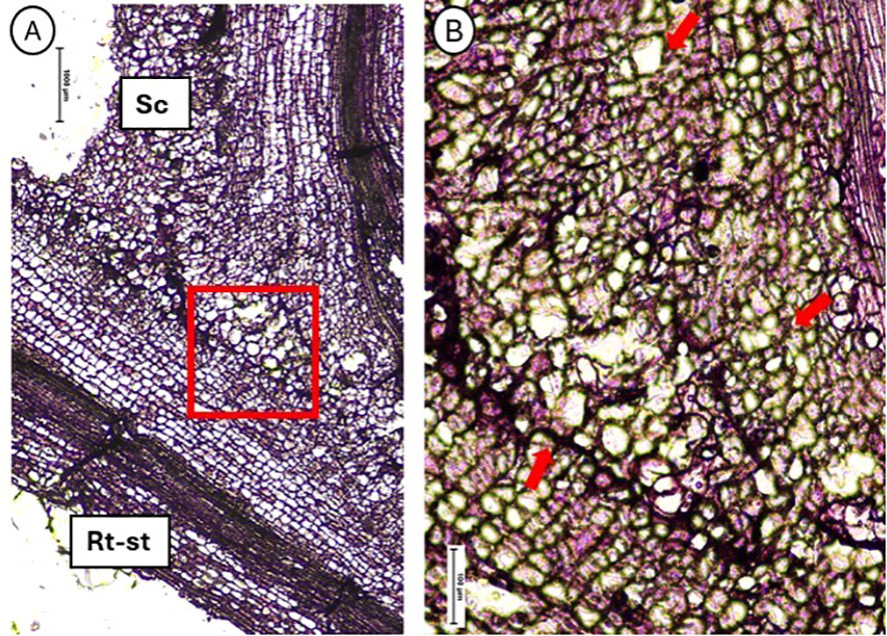
Longitudinal section of a junction area 7 days post IVM between a rootstock (Rt-st) and a scion of Galega (Sc) (homografting). **(A)** Graft interface, including portions of the Sc and the Rt-st. **(B)** Graft junction, highlighting initiation of cell proliferation in the junction area (indicating with red arrows) originating from both the Sc and the Rt-st sides.

**Figure 6:**
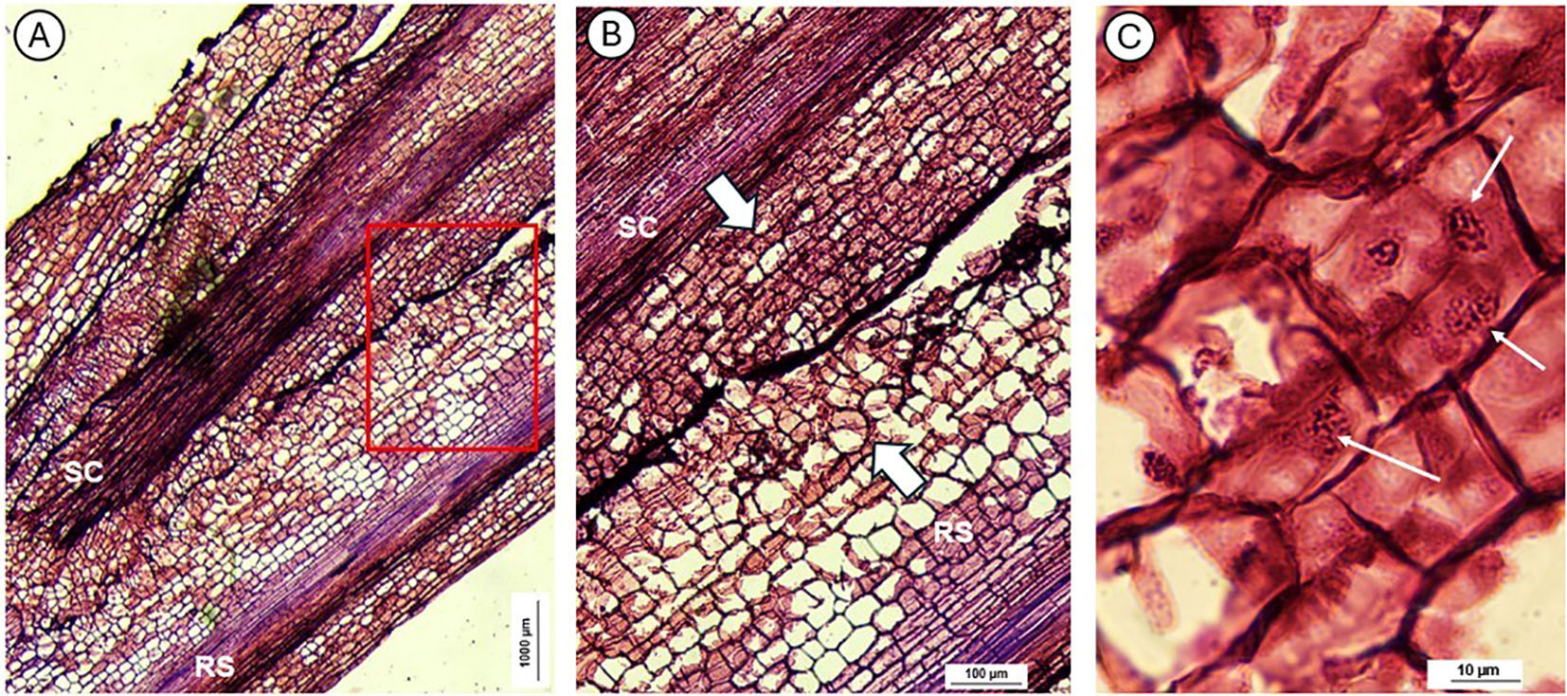
Longitudinal section of a junction area 15 days after micrografting between a rootstock and a scion of Galega (homografting). **(A)** Graft interface, including portions of the scion and the rootstock **(B)** Graft junction, highlighting active cell proliferation originating from both the scion and the rootstock sides. **(C)** Higher magnification (100X) of the graft interface showing cells in mitosis (see arrows), indicating active tissue regeneration.

After the first week, anatomical sections revealed the initiation of callus formation between the scion and the rootstock (**Figures 5A, B**). Observations at two weeks post-IVM showed substantial proliferation of parenchymatous tissue (**Figure 6A**), leading to the formation of callus in both scion and rootstock (**Figure 6B**). Continuous cell division in the parenchyma cells indicated ongoing callus development at the graft union and throughout the lateral regions of both tissues. Evidence of mitotic activity was particularly prominent in the junction zone (**Figure 6C**), contributing to this proliferation.

At this stage, newly formed cambial extensions of the scion and rootstock, which connect to the original cambium of the graft partner, had not yet fused within the callus tissue at the inner surfaces, and a necrotic layer persisted at the graft interface. In successful grafts, the stems of both scion and rootstock maintained their viability, showing a green color, and a distinct callus bridge was established at the graft junction.

**Figure 7:**
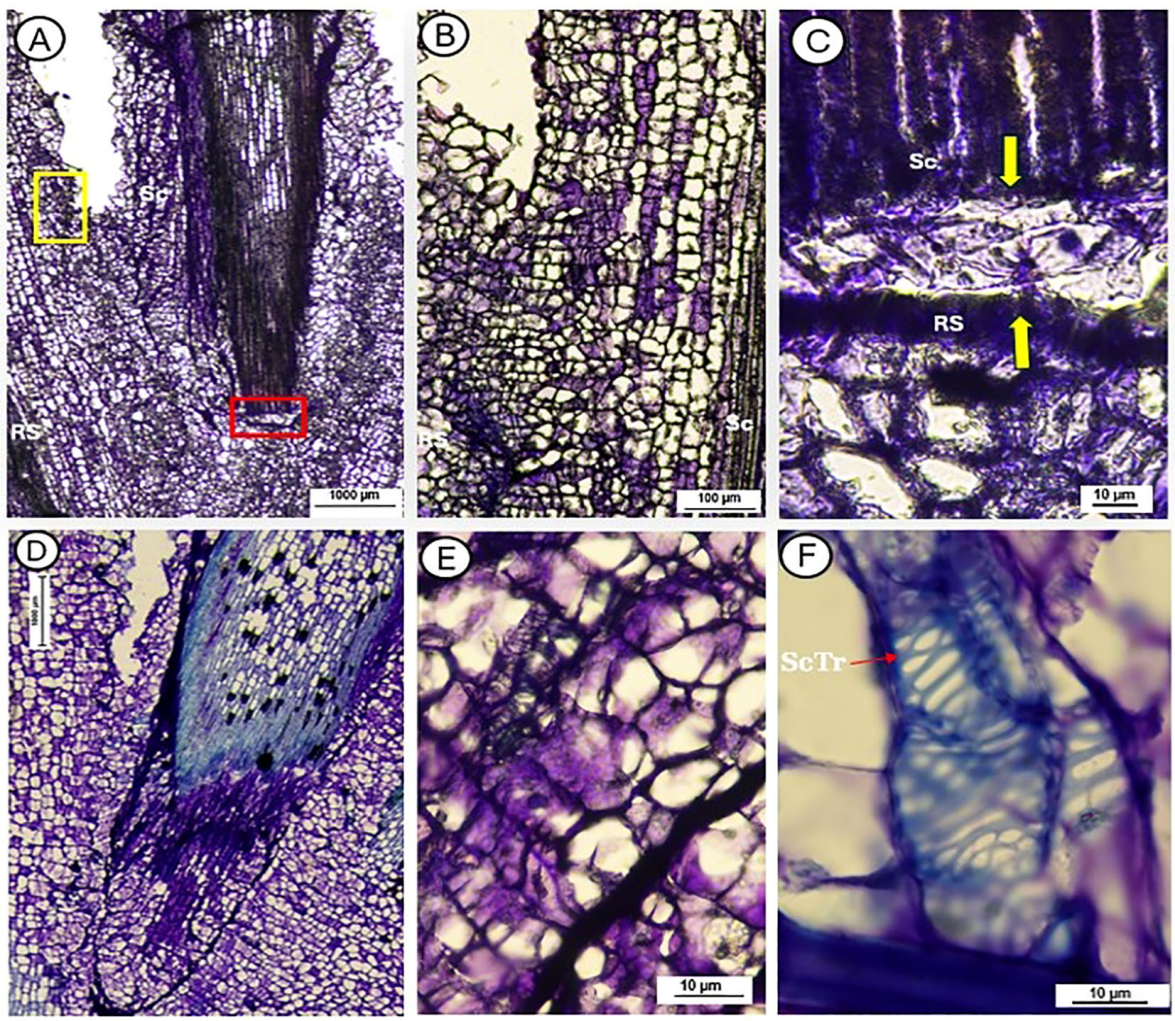
**(A–C)** Histological observations at 21 days after micrografting. Anatomical sections showing cell proliferation in the rootstock near the graft junction. **(A)** Overview of both scion and rootstock regions. **(B)** Lateral region of the graft interface, showing active cell formation. **(C)** Central area of the graft union, highlighting the development of newly formed parenchymatic cells (indicated by yellow arrows), suggesting ongoing callus formation. **(D**, **E)**, **(A, F)** Cell differentiation at the graft junction area at 21 DAM. **(D)** Longitudinal section of the graft union (10X). **(E)** Differentiation of cells in the graft interface into oriented structures, indicative of tissue organization (40X). **(F)** Higher magnification image (100X) showing cells resembling tracheids, suggesting the onset of secondary xylem development.

Cell proliferation and the extension of callus tissue continued progressively in the cross-sections four weeks post-IVM. At this stage, cambial extensions from the scion and rootstock began to connect within the callus tissue, while the necrotic layer at the graft junction started to diminish (**Figures 7A–C**). Cell differentiation at the graft junction area at 21 DAM was observed in **Figures 7D–F**). Oriented structures were observed, indicating tissue reorganization (**Figure 7**). Also at this stage, higher magnification images showed structures resembling tracheid cells, suggesting the re-establishment of secondary xylem development (**Figure 7F**). This phase may serve as a marker for the initiation of vascular continuity between scion and rootstock. In successful grafts, new shoots emerged from the scion, accompanied by a slight increase in scion height.

**Figure 8:**
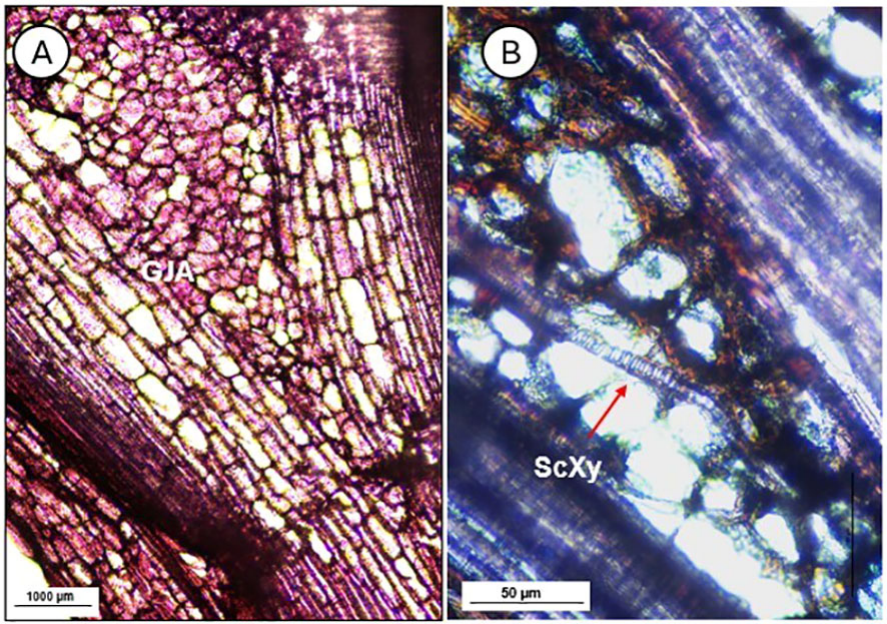
Cellular reorganization at the graft junction area (GJA). **(A)** Longitudinal section of the graft interface showing progressive reduction of cellular asymmetry and early signs of structural alignment and reorganization within the GJA at 35 DAM. **(B)** Lateral section illustrating the formation of a continuous cellular bridge between the scion and rootstock, indicative of a consolidated graft union and advancing vascular integration at 60 DAM.

Microscopic observation 30 days post-IVM revealed the formation of secondary xylem and phloem, with the two types of vascular tissue clearly distinguishable. This stage marked significant progress in establishing contact between scion and rootstock, with reduced asymmetry and initiation of new vascular connections. By the fifth week, cambial formation was clearly observed, with cells becoming more symmetrical and organized, indicating the re-establishment of the vascular connection (**Figures 8A, B**). Throughout this period, scion development was monitored, and cross-sections of the graft samples showed ongoing shoot development, which were attributed to the formation of vascular connections between scion and rootstock that was clearly observed at 60 DAM (**Figure 8B**).

## 4 Discussion

The present study establishes an optimized protocol for *in vitro* ‘V’ grafting using clonal scions and rootstocks in olive. *In vitro* grafting in *Olea europaea* has been investigated since the first work of Revilla et al. (1996), which first demonstrated the feasibility of this technique in olive. Subsequent studies have focused on characterizing graft union development and improving the efficiency and reliability of IVM protocols, largely relying on seedling derived rootstocks to support propagation (Troncoso et al., 1999; Farahani et al., 2011; Vidoy-Mercado et al., 2021). Despite these advances, the use of fully clonal rootstocks in olive micrografting remains largely unexplored.

Grafting can be defined as the natural or deliberate fusion of plant parts as that vascular continuity is established between them (Pina and Errea, 2005), resulting in a genetically composite organism that functions as a single plant. Key factors influencing graft success were identified in the present work, including scions and rootstocks length and diameter. The scion-rootstock length ratio was critical: grafts with scions equal to or shorter than the rootstock achieved the highest success rates, whereas longer scions reduced stability as in our protocol any attachment between the RT and SC was used, likely due to impaired mechanical alignment and reduced physiological integration. Similar observations have been reported in soursop (*Annona muricata*), where scion length and attachment position affected grafting taken, thus survival and growth (Yakubu et al., 2022). This parameter has received limited attention in previous IVM studies, highlighting the applied value of the current protocol. Besides, morphological observations from the first week until 60 DAM provided early indicators of graft success. Callus formation at the junction, maintenance of green tissue in scion and rootstock, as well as emergence of new shoots were consistently associated with successful grafts and considered as a first marker for graft success, while graft failure was characterized by wilting, tissue collapse, and oxidative browning, often linked to early incompatibility and reactive oxygen species (Cookson et al., 2014). Moreover, no significant differences were observed between homo-grafting and hetero-grafting, with clear callus formation distinguishable from 7 DAM. Those observations previously enabled us to select the best way to attach the scion and rootstock in the IVM protocol (Jouini et al., 2024). Other factors such as the composition of the culture medium, the PGRs used, the temperature, and the humidity which are key factors in plant tissue culture can affect the IVM success (Aminzadeh et al., 2013; Akin et al., 2017; Kovalchuk et al., 2018; Davoudi Pahnekolayi et al., 2019). In olive these factors need further investigation and could be the objective of our future directions.

In summary, the combination of callus formation, healthy tissue maintenance, and the emergence of new growth can serve as reliable markers for evaluating the success of grafting in olive and other horticultural practices. Same parameters have been studied in combination with histological analyses as indicators of successful grafting in various species, including *Arabidopsis* (Yin et al., 2012; Melnyk et al., 2015), passionfruit (Ribeiro et al., 2015), *Argania spinosa* (Koufan et al., 2020), Hazelnuts (Balta, 2023), and pecan nuts (Mo et al., 2024). In olive, morphological markers of grafting success have also been investigated in the cultivar ‘Kalamata’ to assess compatibility with different rootstocks (Rashedy and Hamed, 2023).

**Figure 9:**
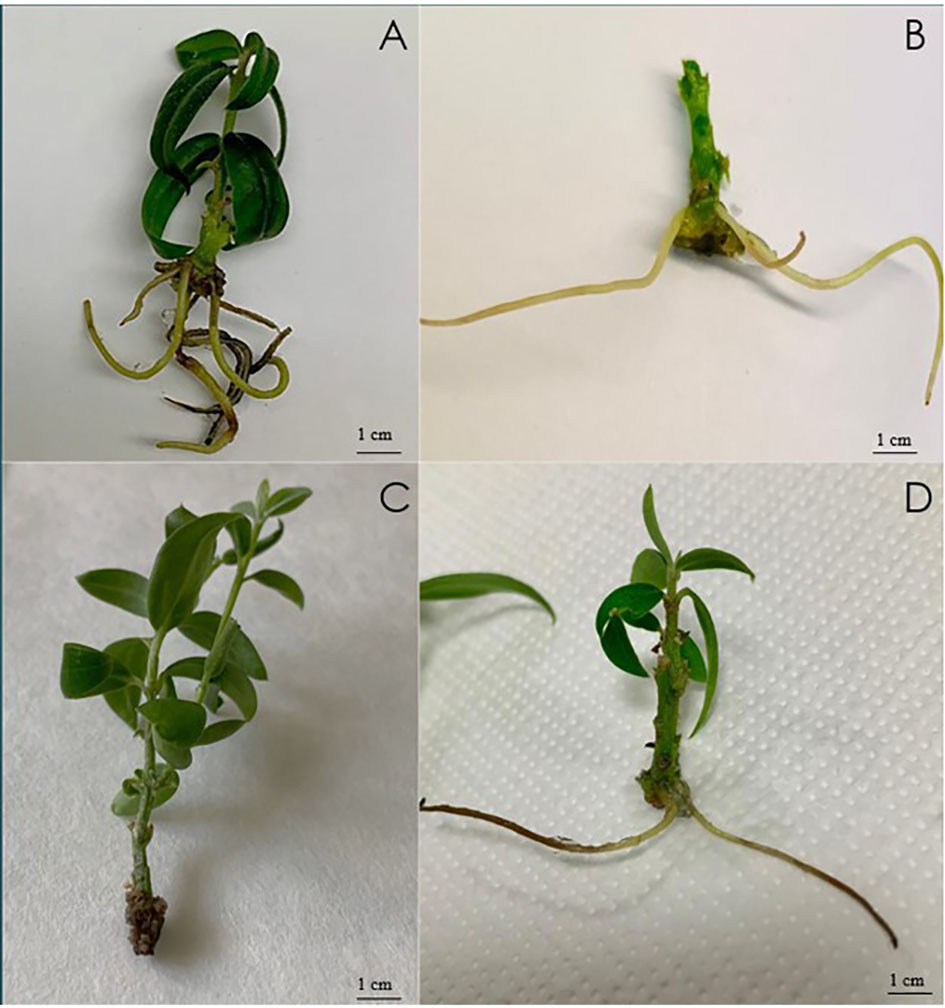
*In vitro* micrografted plants **(A)** Arbequina grafted on rooted rootstock of G35. **(B)** Galega rooted *in vitro* and prepared as a rootstock. **(C)** G26 grafted on Galega rootstock (not rooted). **(D)** G26 grafted on rooted rootstock of G35.

In intact plants, successful graft union formation depends on a complex series of cellular events which include wound response, adhesion, healing in the pith, and the subsequent development of secondary xylem and phloem (Yeoman and Brown, 1976). Graft formation in *Olea europaea* is a complex phenomenon, encompassing several pivotal stages. Our findings from the anatomical observations let us identify the right timing of the different phases of the healing process in olive. Wound response was observed 7 days post-IVM. This preliminary phase is marked by a tremendous/deep cellular proliferation within both the scion and rootstock, characterized by asymmetry, and serves as an early marker of the healing cascade. Callus formation was observed clearly 14 DAM, forming a rigid bridge between the scion and rootstock. This reflects the dynamic nature of tissue regeneration and highlights the importance of callus formation in the healing process from the grafting wound which is a critical phase in establishing a successful graft union (Melnyk et al., 2015). At 15 DAM, histological sections showed proliferation of new cambial extensions from both scion and rootstock, with a necrotic layer persisting at the grafting interface. This callus formation and necrotic layer orientation represent the initial steps of graft union formation, as described by Yeoman and Brown (1976); Pina and Errea (2005), and Tedesco et al. (2022). Alignment and reorganization within the graft junction area were noted at 21 DAM, indicating the formation of new vascular connections. However, the progressive reduction of cellular asymmetry may be a marker for the genesis of new vascular connections, as it was observed together with the differentiation of the cells into tracheid cells. By the 28^th^ DAM, active cell proliferation within the callus tissue remained evident, extensions from the cambium of both scion and rootstock began to interconnect. The necrotic layer at the graft interface was reduced, suggesting the onset of cambial continuity. By the 35^th^ DAM, the newly formed cambium became more defined, with cells exhibiting a symmetric and organized arrangement, indicating ongoing vascular reconnection. Secondary xylem and phloem were clearly observed at 60^th^ DAM, and both vascular tissue types were readily identifiable. Throughout this period, aerial growth coincided with histological evidence of vascular reconnection for both homograft and heterografts, reinforcing the link between anatomical integration and physiological performance. These findings are consistent with Troncoso et al. (1999) who observed initial cellular proliferation for vascular tissue formation 12 DAM in olive, with complete connection achieved by 22 DAM and full graft union established within three weeks. Vigorous plants were subsequently obtained after 60 days of *in vitro* culture followed by 10 days of acclimatization. Evidence from model plants supports this general sequence of healing events. In *Arabidopsis*, graft reconnection occurs as early as 2 DAM, with phloem reconnection preceding xylem reconnection and root growth resumption (Melnyk et al., 2015). In tomato, both homo- and hetero-grafts showed similar development, with thickening of cell walls at the junction and formation of new vascular tissue by 10 DAM (Frey et al., 2020). The healing process was also studied in other fruit species applying IVM (Wang et al., 2022). In almonds, IVM studies have shown that graft type, scion length, and rootstock genotype significantly influence success rates (Yıldırım et al., 2010, Yıldırım et al., 2013; Asadi Zargh Abad and Shekafandeh, 2021). Anatomical studies revealed callus formation around 30 days post-budding and full vascular reconnection within 12 months (Özdemir et al., 2019). Other species display similar dynamics. In passion fruit (*Passiflora edulis*), callus formed within three days post-IVM, followed by procambial differentiation at 10 days, tracheid formation at 15 days, and complete vascular reconnection at 30 days (Ribeiro et al., 2015). In durian, cell division began at day 3, adhesion at day 7, and vascular reconnection between days 30–45, accompanied by changes in phenolic and hormonal profiles (Thippan et al., 2024). Incompatibility can disrupt these processes, as observed in apricot grafted onto different *Prunus* rootstocks, where disorganized cambial tissues were detected in incompatible graft unions (Errea et al., 2001; Pina et al., 2012). These findings support the hypothesis that early cambial disorganization represents a reliable anatomical marker of graft failure. Overall, the literature is consistent with our observations, indicating that successful *in vitro* micrografting depends on multiple factors, including the adoption of an appropriate grafting technique, the compatibility between scion and rootstock genotypes, and the optimization of the scion-to-rootstock size ratio. Beyond the biological findings, the present study also highlights the operational efficiency of IVM as a propagation system for olive at a large scale. The complete experimental framework used to characterize anatomical and morphological changes required 60 days, followed by approximately 10 days of acclimatization, as also reported by Troncoso et al. (1999). However, our results demonstrate that vascular reconnection can be achieved as early as 30 DAM, suggesting that the overall timeline could be further reduced through the optimization of environmental conditions such as humidity, temperature, and light. A key innovation of this study is the use of fully clonal rootstocks propagated and rooted *in vitro* (**Figure 9**), which, to our knowledge, has not been previously reported in olive micrografting systems. This approach offers several advantages, including the elimination of somaclonal variation, a reduction in the duration of the propagation cycle, and the possibility to scale up the multiplication of elite genotypes.

In our protocol, both scions and rootstocks were introduced *in vitro* simultaneously, followed by a multiplication phase of approximately 2–3 months to obtain sufficient plant material. Rootstock candidates were then transferred to a rooting medium for approximately 30 days, after which IVM was performed. Overall, this workflow allows the production of grafted plants ready for acclimatization within approximately 6 months from the initial *in vitro* establishment. Notably, this timeframe could be further reduced when clonal material is already available *in vitro*, and because the system is independent of seasonal constraints. In contrast, conventional nursery protocols to produce seedling-derived rootstocks in Italy may require up to three years before grafting can be performed, and are subject to multiple limiting factors, including biotic and abiotic stresses, seed dormancy, space availability, technical constraints, and lack of uniformity (Fabbri et al., 2005). Therefore, the present approach provides significant added value for both nurseries and breeding programs, offering a rapid, scalable, and standardized propagation system capable of producing uniform plant material and enabling further technological innovation in olive cultivation. Moreover, IVM using clonal rootstocks represents a powerful platform for the early screening of scion–rootstock compatibility, which could contribute to addressing major challenges in olive production, such as canopy size control and the development of rootstocks tolerant or resistant to emerging pathogens like *Xylella fastidiosa*.

## 5 Conclusion

This study demonstrates the feasibility and efficiency of *in vitro* micrografting using clonal rootstocks in olive, providing the first evidence of its successful application within a fully clonal system. The detailed characterization of graft union development establishes a mechanistic framework for understanding the sequential events underlying graft formation, from callus proliferation to vascular reconnection. In addition, these findings support future investigations into the physiological and molecular regulation of graft compatibility and union establishment in olive.

Overall, this approach represents a rapid and reliable propagation strategy, enabling the production of uniform, elite, and potentially disease-resistant plant material. It also offers new opportunities to improve crop performance by enhancing resilience to both abiotic and biotic stresses. Furthermore, the use of specific genotypes, such as ‘G35’, opens the possibility of modulating vegetative vigor through targeted rootstock–scion combinations, providing an additional tool for the optimization of olive cultivation systems.

## Data Availability

The original contributions presented in the study are included in the article/supplementary material. Further inquiries can be directed to the corresponding author.
